# Relationship between pulmonary artery pressure components in patients with advanced heart failure

**DOI:** 10.14814/phy2.70421

**Published:** 2025-06-06

**Authors:** Denis Chemla, Antoine Beurnier, Marc Humbert, David Montani, Philippe Hervé

**Affiliations:** ^1^ Université Paris‐Saclay, School of Medicine Le Kremlin‐Bicêtre France; ^2^ INSERM UMR_S 999 “Pulmonary Hypertension, Pathophysiology and Novel Therapies”, HPPIT, Marie Lannelongue Hospital and Bicêtre Hospital Le Kremlin‐Bicêtre France; ^3^ Assistance Publique – Hôpitaux de Paris (AP‐HP), Department of Physiology – Functional Explorations Bicêtre Hospital Le Kremlin‐Bicêtre France; ^4^ Assistance Publique – Hôpitaux de Paris (AP‐HP), Department of Respiratory and Intensive Care Medicine, Pulmonary Hypertension National Referral Center, FHU André Cournand, ERN‐LUNG Bicêtre Hospital Le Kremlin‐Bicêtre France

To the Editor,

In both normotensive individuals and patients with pulmonary hypertension (PH), systolic (sPAP), diastolic (dPAP), and mean (mPAP) pulmonary artery pressures are strongly correlated. The regression lines describing the sPAP–mPAP relationship are very similar in both pre‐ and post‐capillary PH patients and demonstrate near‐proportionality (Chemla et al., 2004; Miller et al., 2013; Syyed et al., 2008; Vanden Eynden et al., 2016). Although mild differences may arise depending on the underlying cause of PH and the type of catheter used, it is likely that a common pathophysiological mechanism links these pulmonary artery pressure components, whether left ventricular filling pressure is increased, as in post‐capillary PH, or not, as in pre‐capillary PH. A comparable pattern is seen in the mPAP–dPAP relationship, where the correlations remain strong, although somewhat weaker, and dPAP also tends to be slightly higher than expected in post‐capillary PH patients.

Building on these findings, our group proposed that the proportionality constants of the sPAP–mPAP and mPAP–dPAP relationships may approximate the golden ratio in specific patient subgroups (Chemla et al., 2019). The golden ratio (Φ = 1.618) is a special proportion that has been found to appear in many phenomena in nature, from the shapes of plants to the design of certain biological systems, including the cardiovascular field.

We read with great interest the article by Lim & Yim, who investigated our golden ratio hypothesis in the pulmonary circulation of patients with advanced heart failure (HF) due to left‐sided heart diseases being assessed for heart transplantation, or with cardiogenic shock (Lim & Yim, 2025). The authors reported that 12 out of 20 HF patients (60%) and 76 out of 93 patients with cardiogenic shock (82%) fulfilled the hypothesis. The remaining patients deviated from it, as attested to by a lower PP:mPAP ratio, which may help identify patients with more severe haemodynamic compromise. Pulmonary artery pulse pressure (PP) is defined as sPAP – dPAP. We commend Lim & Yim on their proposal and would like to offer a few comments, particularly concerning the HF population.

The authors state that our results were “consistent with the Φ hypothesis in patients with and without PH” (Lim & Yim, 2025). For clarity, our initial proposal applied to 44 normotensive individuals and 981 pre‐capillary PH patients. In that study, we stated that “the mPAP/dPAP ratio may be less strongly constrained around Φ than the sPAP/mPAP (…) especially in post‐capillary patients with PH (unpublished observation)” (Chemla et al., 2019). One hypothesis is that elevated pulmonary artery wedge pressure (PAWP) in post‐capillary PH passively increases dPAP, thereby slightly constraining the mPAP–dPAP relationship, whereas in pre‐capillary PH—where PAWP remains low—dPAP is not subject to the same constraint.

Lim and Yim examined whether the sPAP:mPAP and mPAP:dPAP ratios approximate Φ in HF patients, using high‐fidelity pressure catheters. To account for inevitable measurement errors, they defined a range of 1.46–1.79, which corresponds to an assumed ±5% error in pulmonary artery pressure estimates. This approach is in line with our original proposal, which followed established recommendations in the field (Markowsky, 1992). The range corresponds to Φ ±10%, which appears clinically acceptable.

The authors also focused much of their discussion on the PP:mPAP ratio. While mathematically this ratio should equal 1 if both sPAP and dPAP satisfy the Φ hypothesis, the above‐mentioned method cannot be applied to define a valid range for PP:mPAP. Based on averaged data from ten invasive studies, the authors adopted a range of 0.612–1.228 for this ratio. Given that this range is not mathematically coupled to Φ and appears rather wide, we favor assessing consistency with the Φ hypothesis using the sPAP:mPAP and mPAP:dPAP ratios alone.

Concerning the role of PAWP (as an estimate of left ventricular filling pressure), heart rate, and stroke volume in the context of the Φ hypothesis, it is worth discussing a fluid‐filled catheter study involving a heterogeneous population of 1135 patients, including 332 with HF referred for heart transplantation or left ventricular assist device implantation (Amsallem et al., 2020). In this derivation cohort, an equation incorporating PAWP, heart rate, and age improved the prediction of sPAP from mPAP, while the patient's sex, body surface area, stroke volume and pulmonary vascular resistance did not contribute. The new equation remains to be prospectively validated, as the patient's heart rate was not available in their subsequent so‐called validation cohort. However, compared to our empirical equations relying solely on mPAP (Chemla et al., 2004, 2019), the theoretical improvement in accuracy appears minimal (around 1 mmHg, as calculated from their Figure 4), despite a quadrupling of variables in the model.

On average, the PP:mPAP ratio approaches 1 in pre‐capillary PH (Chemla et al., 2019), reflecting the high pulsatility of pulmonary circulation in these patients. The authors' interpretation of this ratio is thoughtful and interesting (Lim & Yim, 2025). We would like to offer the following additional comments. When interpreting PP values, it is important to consider the mPAP level to distinguish passive increases in PP due to high mPAP (linked to the shape of the stress–strain relationship) from disproportionately high PP, which exceeds expected values for a given mPAP level. The latter mechanism may be mediated by: (i) structural stiffening of the arterial wall: the more rigid the arterial walls, the greater the increase in PP for each increment in mPAP; and (ii) increased stroke volume. As a result, it may be hypothesized that, in a given subgroup of patients, a lower PP:mPAP ratio indicates that their isobaric pulmonary arterial compliance is higher (Chemla et al., 2021), and/or that their stroke volume is lower.

In conclusion, the golden ratio describes how certain pressure measurements in the pulmonary circulation of patients with PH or HF may follow a predictable pattern. Although the golden ratio should not be overstated in pulmonary circulation, whether it reflects merely a fascinating feature or may provide insights into their underlying pathophysiology and have clinical implications remains a potentially important issue. In this respect, Lim & Yim must be congratulated for their highly valuable and appreciated contribution to the field.

## AUTHOR CONTRIBUTIONS

All the authors meet all of the following conditions: drafting the article or revising it critically for important intellectual content; final approval of the version to be published; and agreement to be accountable for all aspects of the work thereby ensuring that questions related to the accuracy or integrity of any part of the work are appropriately investigated and resolved.

## FUNDING INFORMATION

None.

## CONFLICT OF INTEREST STATEMENT

Denis Chemla, Antoine Beurnier, and Philippe Hervé have no COI to declare. Marc Humbert has received consultant fees from 35 Pharma, AOP Orphan Pharmaceuticals, Bayer, Chiesi, Ferrer, Gossamer Bio, Janssen, Keros, Liquidia, MSD, Novartis, Pulmovant, Respira, United Therapeutics Corp, all COIs outside the submitted work. David Montani has received speaker fees from Bayer, Boerhinger, Chiesi, Ferrer, GSK, and MSD; consultancy fees from Boerhinger, Ferrer, and MSD; and research grants from Calcimedica and MSD, all COIs outside the submitted work.
